# Nanogap-enhanced terahertz suppression of superconductivity

**DOI:** 10.1515/nanoph-2025-0487

**Published:** 2025-12-08

**Authors:** Joonyoung Kim, Gangseon Ji, Hyoung-Taek Lee, Jeonghoon Kim, Han-Seok Park, Uksam Choi, Choongwon Seo, Changhee Sohn, Kyungwan Kim, Byeongwon Kang, Hyeong-Ryeol Park

**Affiliations:** Department of Physics, 131639 Ulsan National Institute of Science and Technology (UNIST), Ulsan 44919, Republic of Korea; Department of Mechanical Engineering, Pohang University of Science and Technology (POSTECH), Pohang 37673, Republic of Korea; POSCO-POSTECH-RIST Convergence Research Center for Flat Optics and Metaphotonics, Pohang 37673, Republic of Korea; Department of Physics, Chungbuk National University, Cheongju 28644, Republic of Korea; Linac Coherent Light Source, SLAC National Accelerator Laboratory, Menlo Park, CA 94025, USA; Department of Physics, Graduate School of Semiconductor Materials and Devices Engineering, Graduate School of Health Science and Technology, and Department of Semiconductor Engineering, Ulsan National Institute of Science and Technology (UNIST), Ulsan 44919, Republic of Korea

**Keywords:** superconductivity suppression, terahertz spectroscopy, nanogap, ponderomotive energy, superconducting gap

## Abstract

Superconductivity collapses when all Cooper pairs acquire energies exceeding the superconducting gap. Breaking these pairs requires photons with energy greater than the superconducting gap or strong terahertz (THz) electric fields, which has limited the practical use of superconducting devices at THz frequencies. Here, we show that GdBa_2_Cu_3_O_7-δ_ (GdBCO) film integrated with 15-nm metal nanogaps exhibit Cooper pair breaking at 20 K, which is lower than its critical temperature *T*_c_, under incident THz fields as low as 60 V/cm. It should be noted that the extracted optical constants of the nanogap-integrated film exhibit a characteristic of a non-superconducting state, in contrast to the bare GdBCO film. This suppression of the superconductivity cannot be attributed to heating or fabrication damage but instead arises from the nanogap-enhanced THz fields delivering ponderomotive energy beyond the superconducting gap. Our results establish a non-thermal, low-field pathway for controlling superconductivity, opening opportunities for highly sensitive superconducting optoelectronic devices such as a THz single photon detector.

## 1 Introduction

Superconductors exhibit extremely high electrical conductivity below their critical temperature and possess properties that differ fundamentally from those of conventional metals. Because of these unique features, superconductors have been intensively investigated not only in fundamental physics but also for their potential in large-scale applications such as power transmission, transportation, and industrial systems [1], [2], [3]. In parallel, THz metamaterials, artificially engineered subwavelength resonators, enable exotic responses and intensify light–matter interactions, establishing versatile platforms for sensing, non-destructive inspection and for active tuning when integrated with functional materials [4], [5], [6], [7], [8], [9], [10], [11], [12], [13], [14], [15], [16], [17], [18], [19], [20], [21]. The integration of superconductors with THz metamaterials combines the strengths of both platforms: the conductivity of superconductors varies strongly with temperature, which allows active tuning of the resonance frequency and strength of metamaterial elements [22], [23], [24], [25]. High-*T*_c_ superconducting films, in particular, offer low loss and high-quality factors in the THz regime, and when combined with metamaterials, they can exhibit pronounced switching and modulation of resonances [26], [27], [28]. Such superconducting metamaterials typically exploit temperature control to manipulate superconductivity.

Beyond thermal tuning, superconductivity can also be controlled by electromagnetic excitation. In superconductors, two electrons with opposite spins form a Cooper pair bound by the superconducting gap. When the absorbed energy exceeds this gap, superconductivity collapses, resulting in a sharp increase in direct current (DC) resistance, a principle exploited in devices such as superconducting nanowire single-photon detectors (SNSPDs) [29], [30], [31], [32], [33], [34], [35], [36]. In addition, studies have also shown that intense THz fields can suppress superconductivity, even though their photon energies lie below the superconducting gap [37], [38], [39], [40], [41], [42]. If THz metamaterials could lower the threshold field amplitude required for the superconductivity suppression, the SNSPD concept could be extended into the THz region, enabling highly sensitive detection of weak THz fields, below the current minimum detectable frequency of around 30 THz [43], [44].

**Figure 1:**
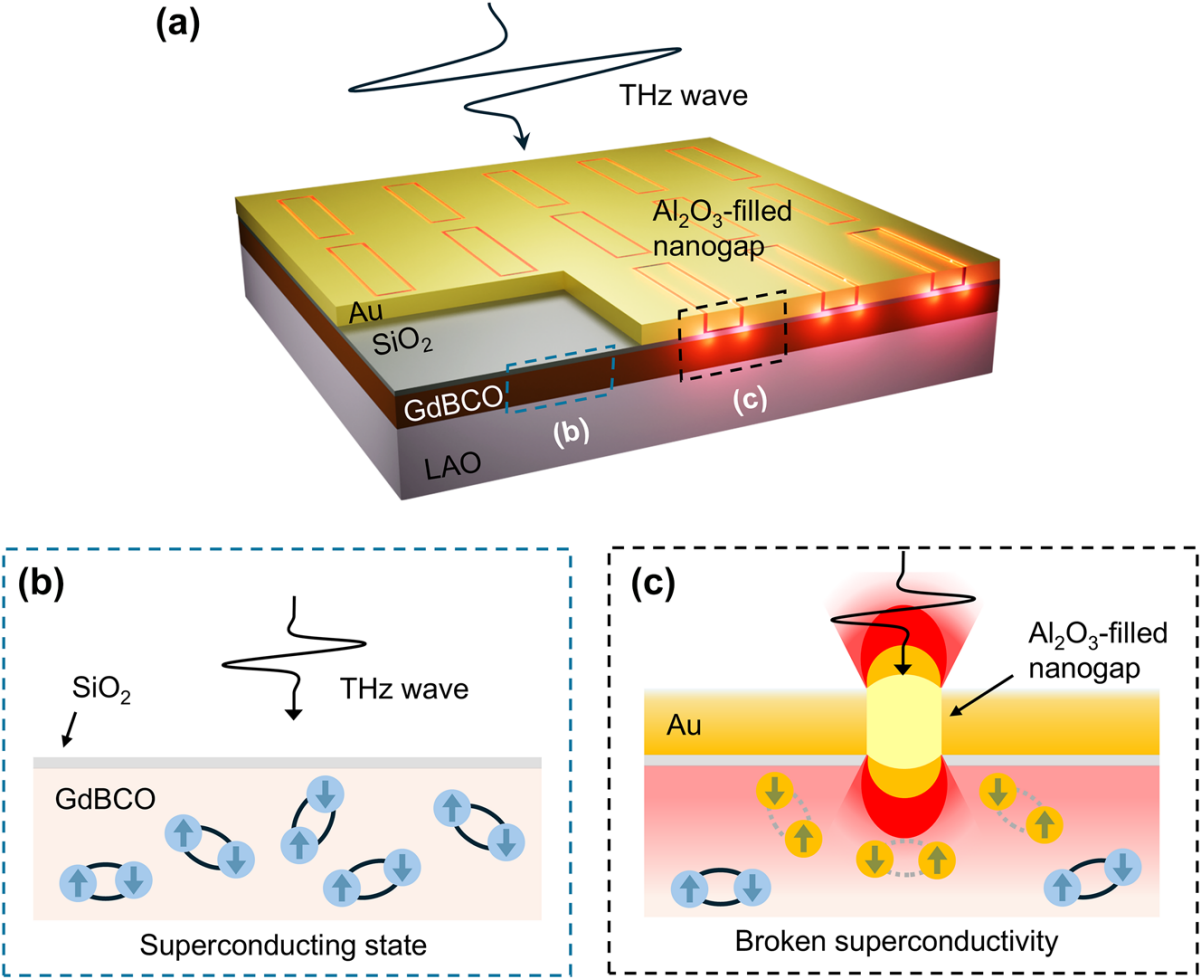
Terahertz-driven superconductivity suppression enhanced by metal nanogap. (a) Schematic of a terahertz wave incident on a GdBCO superconducting film integrated with metal nanogaps (GdBCO nanogap). (b) Illustration of the superconducting state in a region negligibly affected by the incident terahertz field. Blue electrons connected by solid lines represent intact Cooper pairs carrying the supercurrent. (c) Suppression of superconductivity induced by nanogap-enhanced terahertz fields. Yellow electrons connected by dotted lines indicate broken Cooper pairs caused by the terahertz field.

Here, we show that even a THz field as weak as 60 V/cm can suppress superconductivity in a metal nanogap–integrated GdBCO film (hereafter referred to as the GdBCO nanogap), below its superconducting transition temperature (Figure 1(a)). The bare film remains unaffected by the THz field, with optical constants below *T*_c_ = 88 K corresponding to the superconducting state (Figure 1(b)). In contrast, 15 nm-wide metal nanogap array fabricated on the GdBCO film via atomic layer lithography concentrates the incident THz field by several hundred-fold through capacitive charge accumulation at the nanogap edges [45], [46], [47], [48], [49]. This extreme field enhancement drives the underlying GdBCO film into a non-superconducting state (Figure 1(c)), as revealed by the THz transmitted amplitude spectra of the GdBCO nanogap. Finite element analysis (FEA) further confirms that the extracted optical constants correspond to a superconductivity-broken state.

## 2 Results and discussion

**Figure 2:**
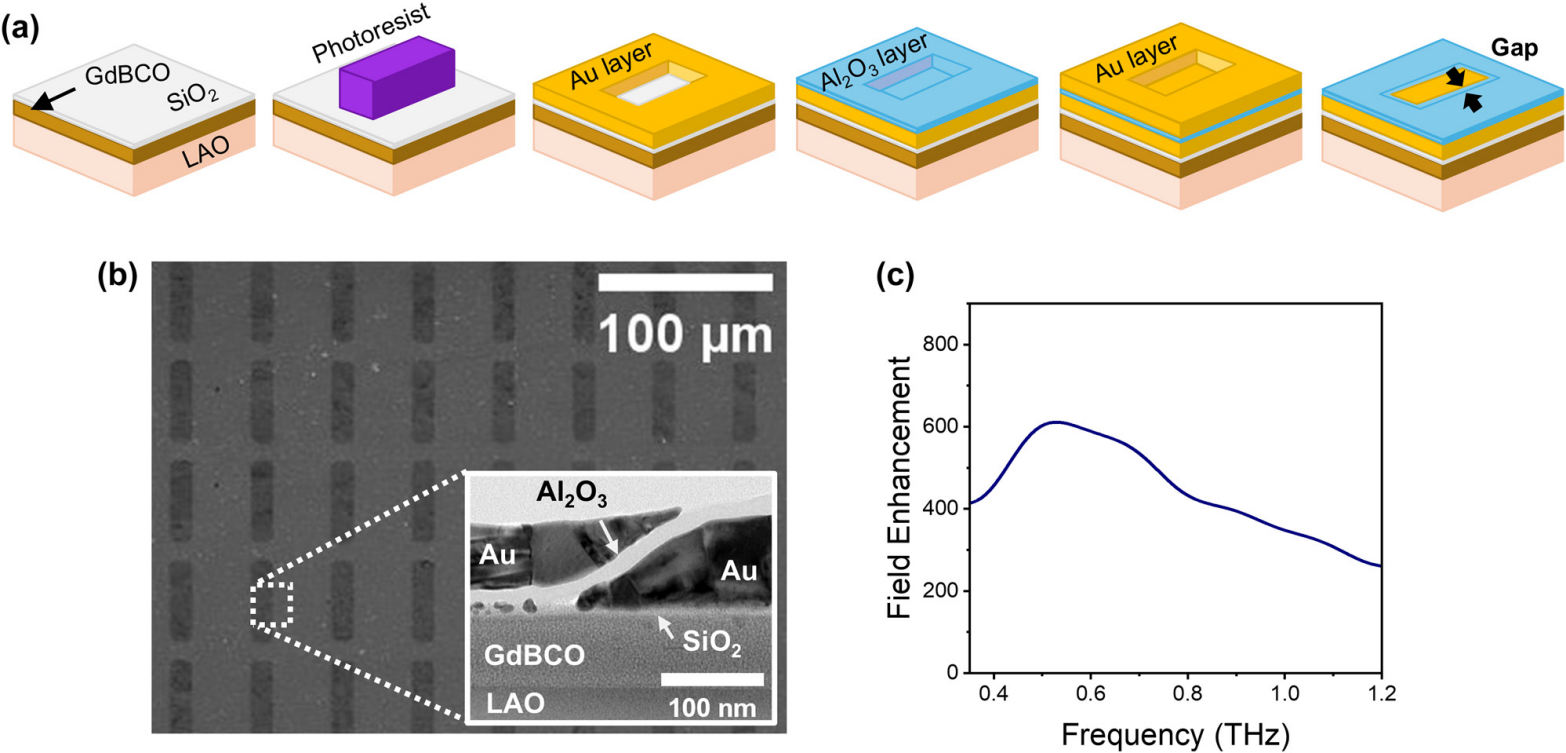
Fabrication and characterization of the GdBCO nanogap. (a) Fabrication process of the GdBCO nanogap using atomic-layer lithography: SiO_2_-capped GdBCO film (bare film), photolithography, first Au deposition (90 nm) followed by lift-off, Al_2_O_3_ atomic layer deposition (15 nm), second Au deposition (70 nm), and peeling-off to form the GdBCO nanogap. (b) Scanning electron microscope (SEM) image of the fabricated nanogap. Inset: cross-sectional transmission electron microscope (TEM) image of a single nanogap, showing a 15 nm-wide Al_2_O_3_-filled gap between two Au layers. (c) Calculated terahertz field-enhancement spectra of the GdBCO nanogap using the Kirchhoff integral formalism, with the maximum enhancement at the resonance frequency of around 0.56 THz.

A 60 nm-thick GdBCO film was grown using pulsed laser deposition, and the GdBCO nanogap sample was subsequently fabricated by atomic layer lithography (see Section 4 and Figure S1 for growth of GdBCO films) [46]. As shown in Figure 2(a), rectangular gold patterns were prepared via photolithography, metal deposition, and lift-off process, respectively. This was followed by the conformal deposition of a 15 nm-thick Al_2_O_3_ spacer layer and the deposition of a second gold layer, after which a peel-off process completed the nanogap structure (see Section 4 for details). Figure 2(b) presents a top-view scanning electron microscope (SEM) image of the fabricated GdBCO nanogap, with the inset showing a cross-sectional transmission electron microscope (TEM) image of a single nanogap. The rectangular pattern array is uniformly arranged with a consistent period. The cross-sectional TEM image reveals a 15 nm-wide Al_2_O_3_-filled nanogap between two Au layers. This nanogap provides the local THz field enhancement. The induced charge generated by the incident THz field accumulates on the Au regions adjacent to the Al_2_O_3_ nanogap, resulting in a locally enhanced THz field [45], [50]. Because the THz field distribution characteristics of the nanogap, including the field enhancement, are minimally affected by the slanted geometry, we employed the Kirchhoff integral formalism to determine the field enhancement. Using this method, the nanogap yields a field enhancement of up to 600 times at the resonant frequency 0.56 THz as shown in Figure 2(c) [48], [51].

**Figure 3:**
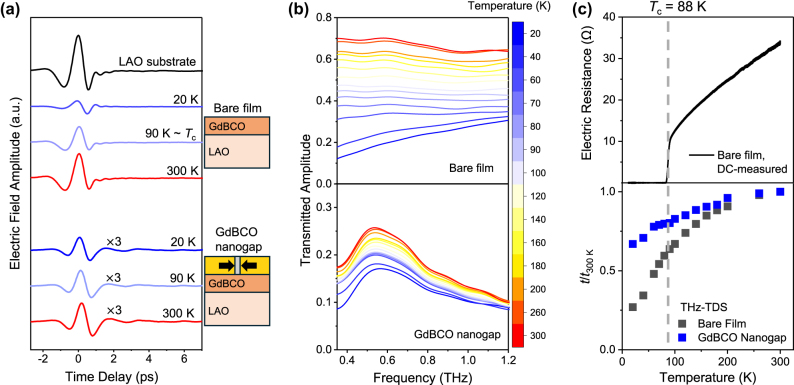
Temperature-dependent terahertz responses of the bare film and the GdBCO nanogap. (a) Terahertz time-domain traces of the bare film and the GdBCO nanogap at various temperatures. Black: LAO substrate (reference); blue: 20 K; light blue: 90 K (near the superconducting transition temperature, *T*_c_ = 88 K); red: 300 K. For the GdBCO nanogap, the pulses are multiplied by a factor of three for clarity. Schematics of each sample are shown next to panel (a). (b) Transmitted amplitude spectra of the bare film (upper panel) and the GdBCO nanogap (lower panel) at different temperatures. (c) DC electrical resistance of the bare film (upper panel) and normalized terahertz transmitted amplitude *t*/*t*_300 K_ at 0.56 THz for the bare film and the GdBCO nanogap (lower panel). The dashed gray line marks *T*_c_ of the bare film.

After fabrication of the GdBCO nanogap, we examined whether the bare film and GdBCO nanogap were in the superconducting or normal state by analysing the optical constants obtained from terahertz time-domain spectroscopy (THz-TDS, see Section 4 and Figure S2 for details). Figure 3(a) shows the transmitted THz pulses through the bare film, the GdBCO nanogap, and a LaAlO_3_ (LAO) substrate as a reference, under an incident THz field of 350 V/cm, in the time domain. For the bare film, the peak electric-field amplitude at 20 K is approximately four times lower than at 300 K. Note that, in contrast to s-wave superconductors with a full gap, cuprate superconductors possess a d-wave gap that leaves low-energy quasiparticles even below *T*_c_. These residual normal carriers give rise to finite THz absorption, allowing measurable transmission through the GdBCO film in the superconducting state [52], [53].

In contrast, for the GdBCO nanogap, the transmitted pulses at 20 K and 300 K have comparable amplitudes. The corresponding transmitted amplitude spectra, obtained via Fourier transform of the time-domain signals in Figure 3(a), are shown in Figure 3(b). As the temperature increases from 20 K to 300 K, the transmitted amplitude of the bare film at the 0.56 THz, the resonance frequency of the nanogap, increases from 0.19 to 0.70, whereas that of the GdBCO nanogap changes only from 0.17 to 0.26. This slight change as temperature decreases, compared to the bare film, provides clear evidence that the superconducting properties are already altered at low temperature in the nanogap-enhanced THz field.

In a conventional superconducting film, the DC conductivity becomes infinite below the superconducting transition temperature (*T*_c_ = 88 K for GdBCO) [54]. At THz frequencies, the optical conductivity remains finite but dramatically increases, reaching values on the order of tens of mΩ^−1^ cm^−1^ below *T*_c_ [37], [55], [56], [57]. The extinction coefficient *κ* characterizes the exponential attenuation of a THz field as it propagates through the material. For a conductor, *κ* can be expressed as [58]:

$$\kappa =\sqrt{\frac{\mu \sigma \omega }{2}}$$

where *μ* is the magnetic permeability, *σ* is the conductivity, and *ω* is the angular frequency of the incident wave. Because *κ* increases with the square root of *σ*, the enhanced conductivity of superconductor at low temperature results in a larger extinction coefficient, leading to a reduced transmitted THz field amplitude [59]. Meanwhile, above *T*_c_, the thin GdBCO film exhibits a much smaller extinction coefficient, giving rise to a higher transmitted amplitude. Consequently, the transition from the low-temperature superconducting state to the high-temperature non-superconducting state yields a pronounced modulation depth in the transmitted amplitude.

For the GdBCO nanogap, however, the transmitted amplitude at 20 K is unexpectedly high, showing little difference compared to that at 300 K. In Figure 3(b), the GdBCO nanogap with gap width of 15 nm exhibits a transmitted amplitude of around 0.17 at the peak frequency of 0.56 THz at 20 K, while a bare film has a transmitted amplitude of approximately 0.19 at 20 K and 0.56 THz. Typically, when a phase transition material is integrated with nanogaps in THz region, the coupling between THz wave and the target material enhances the amplitude modulation between different states, leading to transmission changes even larger than those observed in the bare film [60]. Contrary to this expectation, the measured transmitted amplitude at 20 K is about 0.17, comparable to 0.26 of the GdBCO nanogap at 300 K. This indicates that the GdBCO film beneath the metal nanogap at 20 K is in a state similar to that at 300 K, implying that superconductivity is suppressed even below the critical temperature. It is also noteworthy that, at a lower incident THz field amplitude of 60 V/cm, the GdBCO nanogap exhibits transmitted amplitude spectra similar to those obtained at 350 V/cm in Figure 3(b) (see Figure S3(a) in the Supplementary Material). This further suggests that superconductivity is already broken under the nanogap even at the field amplitude as low as 60 V/cm.

Figure 3(c) presents the DC-measured electrical resistance of the bare film (top panel), along with the normalized THz transmitted amplitude *t*/*t*_300 K_ for both the bare film and the GdBCO nanogap (bottom panel) at 0.56 THz, where *t* is the transmitted amplitude at the temperature of interest, and *t*_300 K_ is the transmitted amplitude at 300 K, used here as a reference. It should be noted that the *t*/*t*_300 K_ for the GdBCO nanogap at 20 K remains anomalously high, comparable to that of the bare film at 100 K, which lies above the superconducting transition temperature, *T*_c_.

**Figure 4:**
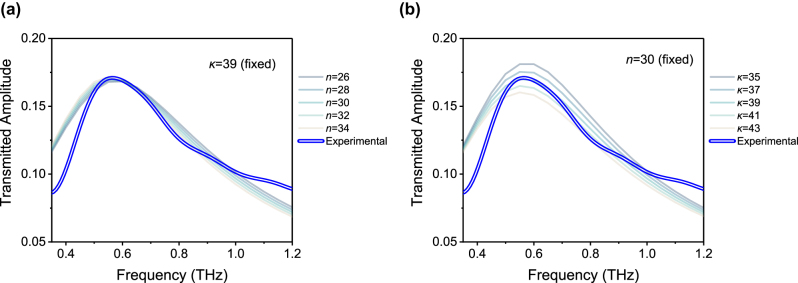
Estimation of refractive index *n* and extinction coefficient *κ* using COMSOL simulations. (a) Parameter sweep of the refractive index of the GdBCO film to reproduce the 0.56 THz resonance of the GdBCO nanogap. (b) Parameter sweep of the extinction coefficient of the film to match the transmitted amplitude of the nanogap. During each sweep, the other parameter was kept constant. The blue double line represents the experimental result of the GdBCO nanogap at 20 K under an incident field of 350 V/cm.

To further understand the transmitted amplitude spectra of the GdBCO nanogap at 20 K, it is necessary that the optical constants, specifically, the refractive index *n* and extinction coefficient *κ*, of the GdBCO film combined with the nanogap is investigated. We used the wave optics module in COMSOL Multiphysics to estimate the effective optical constants of the GdBCO film underneath the nanogap. A simulation domain replicating the real nanogap structure (see Section 4 for the details of finite element analysis) was constructed, and the transmitted amplitude spectra were calculated by sweeping the incident frequency. First, the refractive index was adjusted to align the resonance frequency with the experimental data (Figure 4(a)), followed by tuning the extinction coefficient to match the transmitted amplitude (Figure 4(b)). This iterative procedure was performed until the simulated spectra closely reproduced the experimental results. All these procedures were also repeated to extract the optical constants of the GdBCO nanogap at THz field amplitude 60 V/cm, and 28 V/cm.

**Figure 5:**
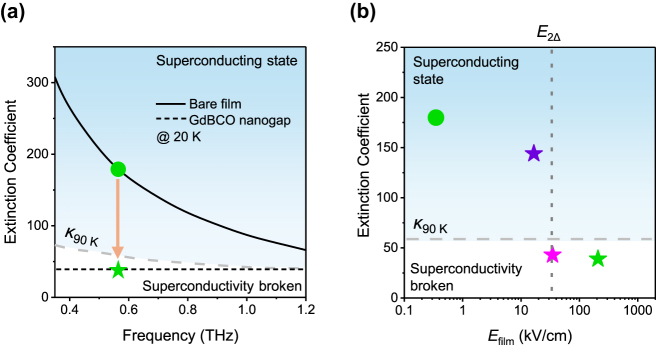
Terahertz field-dependent extinction coefficient and superconductivity suppression in the GdBCO nanogap. (a) Extinction coefficient spectra of the bare film and the GdBCO nanogap at 20 K. (b) Extinction coefficient of the bare film and the nanogap at 20 K and 0.56 THz as a function of the THz field experienced by the GdBCO film, *E*_film_. The dashed gray lines in (a) and (b) indicates the extinction coefficient of the bare film at 90 K. The dotted black line in (a) extends the extinction coefficient at 0.56 THz to other frequencies. The dotted dark-gray line in (b) marks the threshold field *E*_2Δ_ at which the ponderomotive energy equals the superconducting gap 2Δ. Symbols: circles, bare film; stars, GdBCO nanogap. Green data points correspond to an incident field of *E*_inc_ = 350 V/cm, the magenta data point corresponds to *E*_inc_ = 60 V/cm, and the violet data point corresponds to *E*_inc_ = 28 V/cm.

Figure 5(a) shows the extinction coefficient spectra of the bare film and the GdBCO nanogap at 20 K. The extinction coefficient of the bare film, calculated from the complex transmitted amplitude spectra [61], is plotted as a solid black line, with its value at the nanogap resonance frequency highlighted by the green circle. For the GdBCO film underneath the nanogap, the representative extinction coefficient at the same resonance frequency (green star, extracted as described in Figure 4) is extended across the spectrum as a dotted black line. Although the full spectral dependence for the GdBCO nanogap cannot be calculated due to its inherent resonant property, the extinction coefficient of the GdBCO film affected by nanogap-enhanced THz field is significantly smaller than that of the bare film, even lower than the extinction coefficient of the bare film at 90 K (*κ*_90 K_). Since *κ* generally increases monotonically as temperature decreases in superconductors (see Figure S4), *κ*_90 K_ provides a useful reference boundary: values below this line indicate that superconductivity is likely suppressed, whereas those above suggest that the superconducting state is preserved.

Figure 5(b) shows the THz field experienced by the GdBCO film, *E*_film_, together with the corresponding extinction coefficients. In our THz-TDS system, the incident THz field on the sample is 350 V/cm, which is directly experienced by the bare film (green circle). In contrast, the film underneath the nanogap experiences a locally enhanced electric field, with the corresponding extinction coefficient plotted as the green star, being consistent with Figure 5(a). The pink star represents the nanogap under an incident THz field of 60 V/cm. The violet star represents the nanogap under an incident THz field of 28 V/cm, with the GdBCO film thickness of 90 nm for the confirmation of superconductivity maintaining under low THz field. When compared against the reference line of *κ*_90 K_ introduced in Figure 5(a), these data clearly show that superconductivity combined with the nanogap is suppressed even under the incident field of 60 V/cm but not suppressed under the field of 28 V/cm.

Comparing the two green circle and star points in Figure 5(b), which correspond to the same incident THz field, the bare film experiences less than 1 kV/cm, whereas the GdBCO under the nanogap experiences on the order of several hundred times greater due to strong field enhancement near the nanogap. In superconductors, Cooper pairs, which carry the supercurrent, can be disrupted if they acquire energy exceeding the superconducting gap 2Δ [29], [30], [31], [34], [62]. Under incident THz field *E*, Cooper pairs gain ponderomotive energy 2*U*_*p*_:

$$2U_{p}=\frac{e^{2}E^{2}}{2m\omega^{2}}$$

where *e* is the electron charge, *m* is the electron mass, and *ω* is the angular frequency of the THz field [38], [41], [42]. Superconductivity is broken when 2*U*_*p*_ > 2Δ.

For GdBCO, with 2Δ ≈ 50 meV at the temperature 20 K [63] and the dominant frequency in our system *ω*/2*π* = 0.51 THz (see Figure S2(c) to check the spectrum of our THz-TDS system), the threshold field at which the ponderomotive energy just exceeds the superconducting gap is given by

$$E_{2\Delta }=\frac{2\omega }{e}\sqrt{m\Delta }$$

yielding a value of *E*_2Δ_ ≈ 34 kV/cm. For the bare film, the incident field has much smaller than *E*_2Δ_, so the superconducting state is maintained as described in Figure 1(b). For the GdBCO nanogap, however, the enhanced field in the GdBCO nanogap substantially exceeds *E*_2Δ_, thereby breaking Cooper pairs and suppressing superconductivity as described in Figure 1(c). Even when the incident field is reduced to about 60 V/cm, the nanogap-enhanced field still surpasses *E*_2Δ_, as indicated by the pink star in Figure 5(b), demonstrating that this lower external field is also sufficient to break superconductivity. We further examined the superconducting state using another GdBCO nanogap fabricated with a 90-nm-thick GdBCO film and measured with a THz-TDS system driven by an oscillator laser, which generates a weaker THz field of 28 V/cm (see Figure S3(b) to check the transmitted amplitude spectra for the 90 nm-GdBCO nanogap). From this measurement, we confirmed that the enhanced local THz field is approximately 17 kV/cm which is below *E*_2Δ_ and therefore the superconductivity of the GdBCO film is preserved as indicated by the violet star in Figure 5(b). We also confirm that heating due to the applied THz field is insufficient to raise the film temperature to *T*_c_ [37], [38], [39], [41]. Furthermore, the observed change is independent of any degradation of the GdBCO film during the fabrication processes (see Figure S5 for details), confirming that the superconductivity breaking originates from the nanogap-enhanced THz field.

## 3 Conclusions

We demonstrated that nanogap structures enable control of superconductivity in GdBCO films using THz fields. These THz fields have energies below the superconducting gap and relatively small amplitudes. Using transmission-type THz-TDS, we measured the optical constants of the bare film, representing the unsuppressed superconducting state. When a nanogap array was integrated on the film, the retrieved optical constants at cryogenic temperatures were found to be consistent with those of the bare film in the non-superconducting state above *T*_c_, even when the incident field was reduced to as low as 60 V/cm. This behavior cannot be attributed to thermal effects or film degradation during fabrication, but instead arises from the nanogap-enhanced local THz field, which delivers ponderomotive energy to Cooper pairs that exceeds the superconducting gap. Our findings suggest that by further optimizing nanogap parameters such as periodicity, pattern size, and nanogap width using deep learning algorithms, superconductivity could be disrupted with even weaker incident fields [48]. This study opens a pathway to leveraging superconductors to detect weak THz fields that have until now been difficult to access, potentially enabling next-generation high-frequency communication, quantum sensing, and THz imaging.

## 4 Methods

### 4.1 Sample fabrication

A GdBCO superconducting sample was fabricated by epitaxially depositing a GdBCO film on a 0.5 mm-thick (001)-oriented LaAlO_3_ substrate using pulsed laser deposition. The film surface was subsequently planarized, and its thickness was adjusted to 60 nm via Ar ion milling. To prevent chemical degradation during fabrication and measurement processes, the GdBCO film was capped with a 10 nm-thick SiO_2_ layer. On top of this SiO_2_-capped film, atomic layer lithography was employed to define 15 nm-wide gold nanogaps. Rectangular patterns of 10 μm × 40 μm, arranged with a periodicity of 40 μm × 50 μm, were formed using photolithography with a SUSS MicroTec MA6 mask aligner and AZ nLOF 2035 photoresist. A 5-nm-thick chromium layer to enhance adhesion between the gold and the underlying superconducting film was deposited using a Temescal FC-2000 E-beam evaporator, preceded by a 90 nm-thick gold layer. The photoresist and overlying metal were then removed using N-methyl-2-pyrrolidone (NMP), resulting in rectangular metal hole arrays. 15 nm-thick conformal Al_2_O_3_ layer was subsequently deposited onto the rectangular hole array structure via atomic layer deposition (ALD) using an NCD Lucida D100, forming the dielectric spacer that would become the nanogap between two gold layers. After the ALD process, a 70 nm-thick gold layer was deposited, and the excess gold was removed using ion milling followed by a tape-assisted peel-off process. This procedure yields an Al_2_O_3_-filled 15 nm nanogap between adjacent gold layers, forming the final GdBCO nanogap structure. The overall procedure was repeated identically for the fabrication of GdBCO nanogaps using a 90-nm-thick GdBCO film, in order to confirm that superconductivity is maintained under THz fields lower than *E*_2Δ_.

### 4.2 Terahertz time-domain spectroscopy

The optical properties of the GdBCO superconducting film and nanogap were characterized using THz-TDS. THz pulses were generated by a GaAs photoconductive antenna excited by two different ultrashort laser pulses (see Figure S2 for detail). The peak electric field of the THz pulse was approximately 350 V/cm when OPCPA was used. The THz pulses were detected via electro-optic sampling with a 1-mm-thick ZnTe (110) crystal. The THz time transients were Fourier-transformed to obtain the THz spectra of the samples. The samples were mounted in a closed-cycle helium cryostat for the temperature-dependent measurements. As described in Figure S2(a), the measurement chamber was purged using N_2_ gas to minimize water vapor absorption of THz pulses.

### 4.3 Finite element analysis

We employed a commercial finite element analysis tool, COMSOL Multiphysics, to extract the extinction coefficients of the GdBCO film beneath the metal nanogaps. The simulation domain was constructed to mimic the experimental structure, consisting of 15 nm-wide Al_2_O_3_-filled gold nanogaps (refractive index of Al_2_O_3_ = 2.35 + 0*i* for its 20 nm thickness [46]) with a rectangular pattern size of 10 µm × 40 µm, a periodicity of 40 µm × 50 µm, and a thickness of 90 nm. These nanogaps were positioned on a layered structure comprising a 10 nm-thick SiO_2_ capping layer with refractive index of 2 + 0*i*, a 90 nm-thick GdBCO film, and a semi-infinite LAO substrate (refractive index = 4.8 + 0*i*). The refractive index of Au layer was calculated using Drude model, expressed as

$$\tilde{\varepsilon }\left(\omega \right)=\varepsilon_{\infty }-\frac{\omega_{p}^{2}}{\omega^{2}+i\omega \omega_{\tau }}$$

where the Drude parameters for gold are *ω*_*p*_ = 1.37 × 10^4^ THz, *ω*_*τ*_ = 6.78 THz, and *ε*_∞_ = 10 [64]. The value of *ω*_*τ*_ was set to be smaller than its room-temperature value to account for the increased conductivity of the metal at low temperatures [65], [66]. All materials in this simulation were assumed to be isotropic, meaning that all diagonal elements of the permittivity tensor are equal and all off-diagonal components are zero. Perfectly matched layers (PMLs) were applied to the top and bottom of the domain to eliminate undesired reflections and diffractions. To reduce the computational load, symmetry boundaries were used according to the periodicity of the structure. The incident THz pulse was modelled with normal incidence (0°) and polarization perpendicular to the longer axis of the nanogap. The transmitted amplitude spectra were calculated, and the simulation was iteratively repeated while sweeping the complex refractive index of the GdBCO layer to match the simulated transmitted amplitude spectra with the experimental results.

## Supplementary Material

Supplementary Material Details
