# Supplementary material for: Nanogap-enhanced terahertz suppression of superconductivity
Source: Nanophotonics. 2025 Dec 8;14(27):5485–94. doi: 10.1515/nanoph-2025-0487 (PMC12717914; doi:10.1515/nanoph-2025-0487)
Supplement: Supplementary file 1 — Supplementary Material Details [file j_nanoph-2025-0487_suppl_001.pdf]

Supporting Information

Nanogap-Enhanced Terahertz Suppression of Superconductivity

*Joonyoung Kim[1], Gangseon Ji[1], Hyoung-Taek Lee[1,2,3], Jeonghoon Kim[1], Han-Seok Park[4], Uksam Choi[1], Choongwon Seo[5], Changhee Sohn[1], Kyungwan Kim[4], Byeongwon Kang[4], Hyeong-Ryeol Park[1,6]**

[1]*Department of Physics, Ulsan National Institute of Science and Technology (UNIST), Ulsan 44919, Republic of Korea*

[2]*Department of Mechanical Engineering, Pohang University of Science and Technology (POSTECH), Pohang, 37673, Republic of Korea,*

[3]*POSCO-POSTECH-RIST Convergence Research Center for Flat Optics and Metaphotonics, Pohang, 37673, Republic of Korea*

[4]*Department of Physics, Chungbuk National University, Cheongju 28644, Republic of Korea*

[5]*Linac Coherent Light Source, SLAC National Accelerator Laboratory, Menlo Park, CA 94025, USA*

[6]*Graduate School of Semiconductor Materials and Devices Engineering, Graduate School of Health Science and Technology, and Department of Semiconductor Engineering, UNIST, Ulsan 44919, Republic of Korea*

E-mail: *nano@unist.ac.kr

1. Characteristics of the GdBCO film

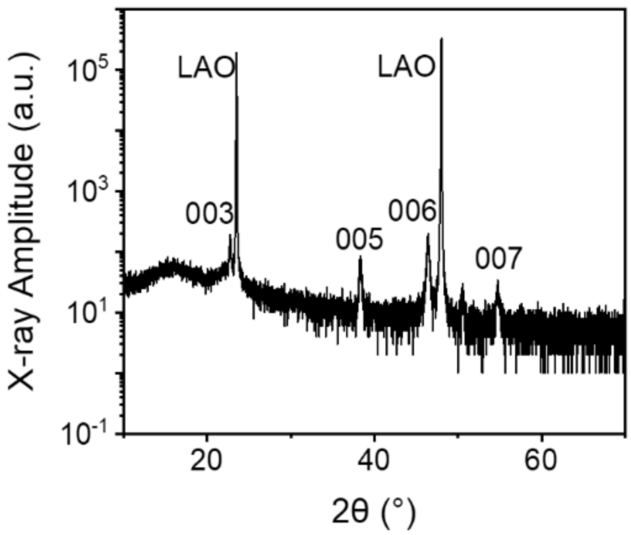

Figure S1. Characteristics of the 60 nm-thick GdBCO bare film: X-ray diffraction (XRD) pattern. The GdBCO peaks appear at 22.75° (003), 38.33° (005), 46.47° (006), and 54.75° (007). See Figure 3(c) to check the electrical characteristics and the superconducting transition temperature of the GdBCO film.

2. Characterization of the THz-TDS system

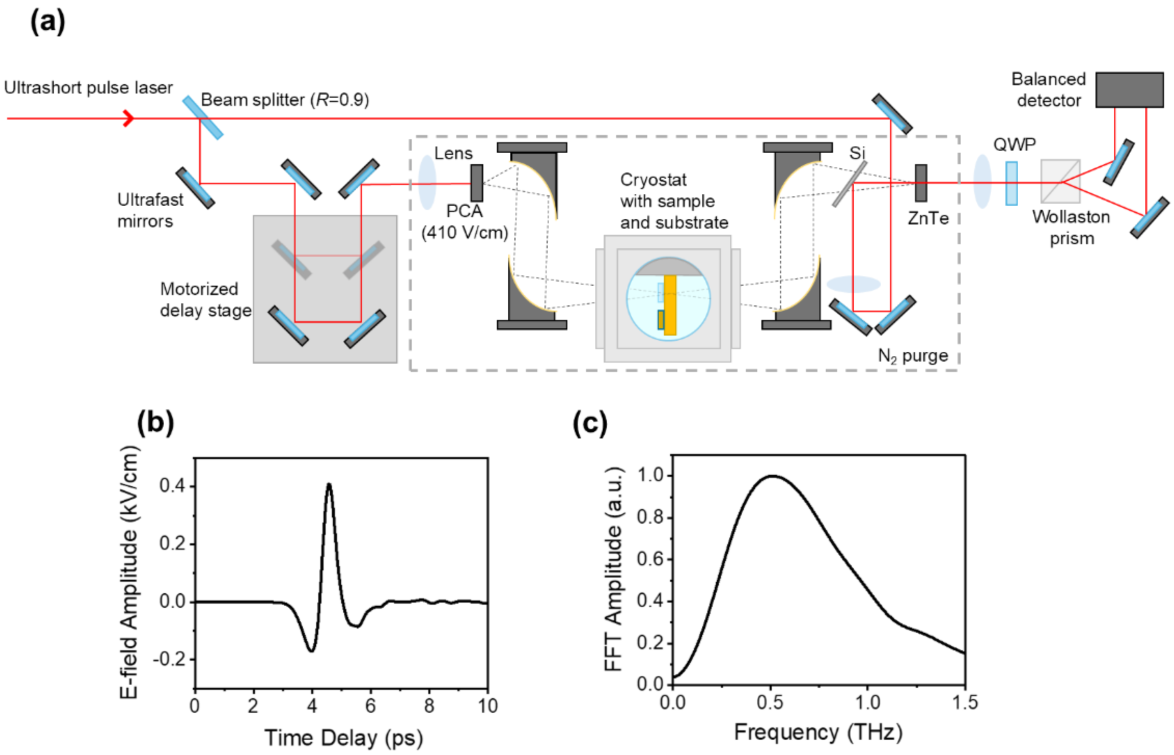

Figure S2. (a) Schematic of the low-temperature terahertz time-domain spectroscopy (THz-TDS) setup. Two different ultrashort-pulse laser systems were used: an OPCPA laser (pulse width < 10 fs, center wavelength 800 nm, pulse energy 1.1 μJ, repetition rate 1 MHz) and an oscillator laser (pulse width < 115 fs, center wavelength 800 nm, pulse energy 13 nJ, repetition rate 80 MHz). (b) Time trace of the THz pulse measured at the focal point without the cryostat when the OPCPA laser is used. The peak electric-field amplitude is reduced to 350 V/cm by placing a z-cut quartz window in front of the sample. (c) Fourier-transformed amplitude spectrum corresponding to (b), showing a maximum around 0.51 THz.

3. Terahertz transmitted amplitude spectra for the GdBCO nanogap at incident E-fields of 60 V/cm and 28 V/cm.

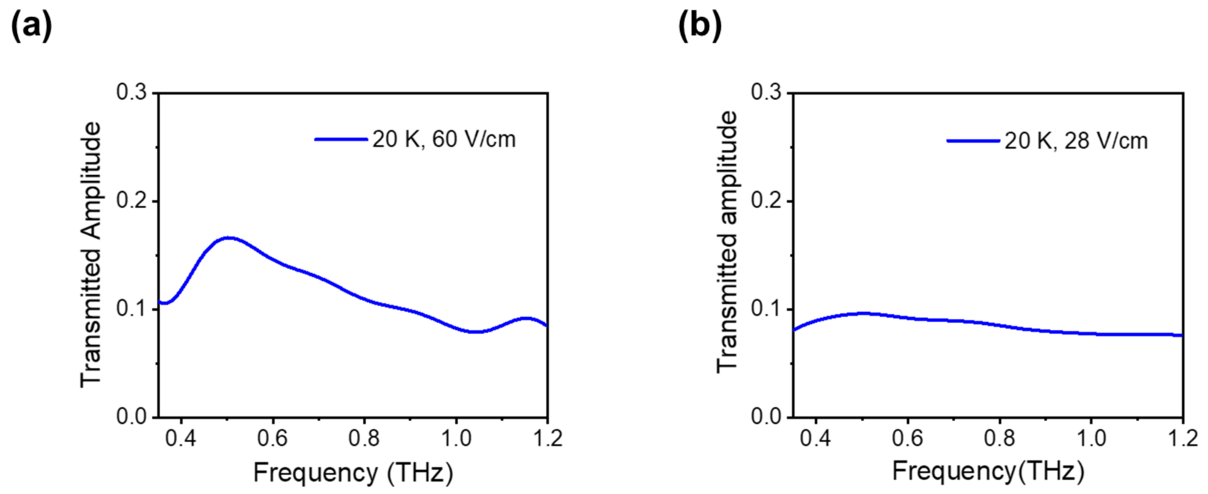

Figure S3. Transmitted amplitude spectra of the GdBCO nanogap under incident fields of (a) 60 V/cm and (b) 28 V/cm. Two transmitted amplitudes are measured at the temperature of 20 K.

4. Extinction coefficient of the GdBCO bare film.

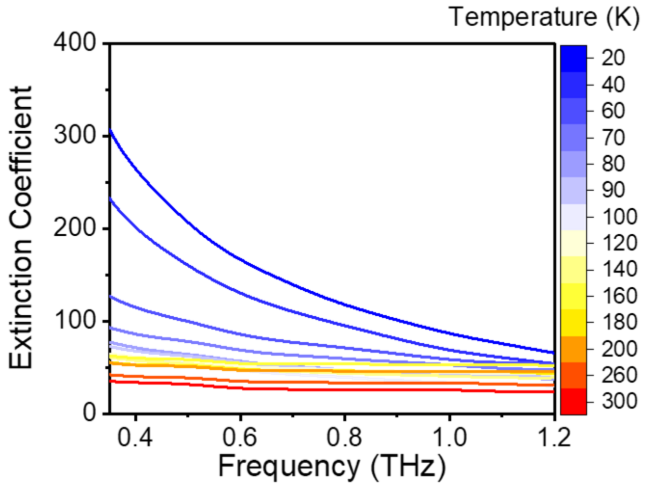

Figure S4. Extinction coefficient spectra of the bare film at various temperatures. The extinction coefficient decreases monotonically with increasing temperature.

5. Superconductivity not suppressed by fabrication of metal structures

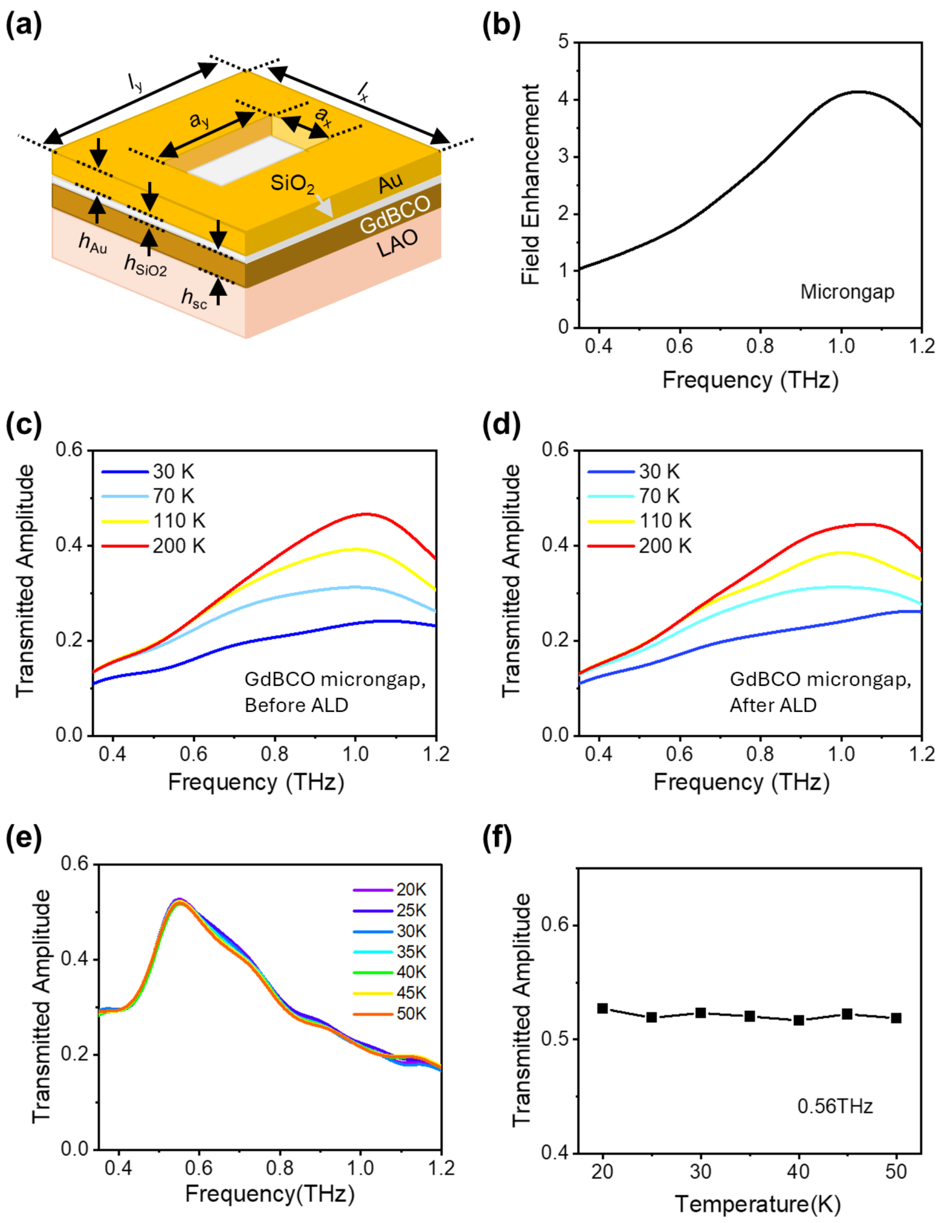

Figure S5. (a) Scheme of the GdBCO film integrated with a rectangular metal hole pattern (GdBCO microngap). (b) THz field enhancement spectrum for the GdBCO mirongap. (c,d) Transmitted amplitude spectra of the GdBCO microngap at various temperatures: (c) before and (d) after Al_2O_3 deposition by atomic layer deposition. (e) Transmitted amplitude spectra of the GdBCO nanogap without the SiO_2 capping layer at different temperatures. (f) Transmitted amplitude at the resonance frequency of 0.56 THz for the uncapped nanogap.

The GdBCO film integrated with a rectangular metal hole pattern (GdBCO microngap) was fabricated using the same procedure as for the GdBCO nanogap, up to the NMP lift-off step (see Methods for details). The resulting GdBCO microngap as shown in Figure S5(a) has a_x=10 μm, a_y=40 μm, l_x=40 μm, l_y=50 μm, h_{Au}=90 nm, h_{SiO2}=10 nm, and h_{sc}=60 nm. For the GdBCO microngap, the THz field enhancement is about 4 as shown in Figure S5(b), corresponding to an effective field of E = 4×350 V/cm = 1.4 kV/cm, which is smaller than $E_{2\Delta}$. Thus, the microngap-enhanced field is insufficient to suppress superconductivity, and the GdBCO film is expected to remain superconducting below its transition temperature (T_c).

To further confirm that superconductivity was preserved during the thin-film deposition of Al_2O_3, we performed temperature-dependent THz transmission measurements on the GdBCO microngap sample before and after the atomic layer deposition (ALD) process. The integrated Al_2O_3 layer does not substantially alter the transmitted THz amplitude, due to its relatively low refractive index compared with that of the GdBCO film and Au layer. This negligible influence is verified by the transmitted amplitude spectra in Figures S5(c) and S5(d), which demonstrate nearly identical temperature-dependent modulation before and after Al_2O_3 deposition. Therefore, when the GdBCO film was protected by a thin SiO_2 capping layer, superconductivity was maintained during the ALD of Al_2O_3, and the transmitted amplitude modulation remained comparable to that of the bare GdBCO film.

If the GdBCO nanogap is fabricated without the SiO_2 capping layer, no temperature-dependent modulation of the transmitted amplitude is observed, as shown in Figure S5(e) and S5(f). Even though superconductivity is suppressed within the nanogap, modulation comparable to that of the bare film in the non-superconducting state, as shown as Figure 3(c), would still be expected as shown as Figure 3(c), but it is absent. These results indicate that the SiO_2 capping layer is essential for protecting the GdBCO film and preserving its superconductivity [1]. During the ALD process of Al_2O_3, the oxygen stoichiometry of the superconducting film can be altered, leading to chemical modification and degradation of superconductivity. The SiO_2 capping layer therefore plays a critical role in preventing such chemical changes, while its thickness was minimized to reduce the separation between the nanogap and the film and to limit its influence on the optical response.

References

[1] J. Du, K. Leslie, C. Foley, G. Harding, B. Sankrithyan, and D. Tilbrook, "Effects of sputtered SiO_2 passivation layers on YBCO microbridges and step-edge junctions," *Supercond. Sci. Technol.,* vol. 12, no. 11, p. 1027, 1999, doi: https://doi.org/10.1088/0953-2048/12/11/399.
